# A surgically treated case of severe upper gastrointestinal hemorrhage with gastritis cystica polyposa

**DOI:** 10.1186/s12876-020-01595-3

**Published:** 2021-01-13

**Authors:** Masaaki Yoshikawa, Hiroki Kinoshita, Naoki Nishimura, Rieko Takai, Takuya Matsuda, Satoshi Nakatani, Erika Shioyama, Kosuke Takeda, Hitoshi Yoshiji

**Affiliations:** 1 Department of Internal Medicine, Kokuho Central Hospital, 404-1 Miyako, Tawaramoto, Nara Prefecture 636-0302 Japan; 2 Department of Gastroenterology, Nara Medical University, Kashihara, Nara Prefecture 634-8522 Japan

**Keywords:** Gastritis cystica polyposa, Upper-GI hemorrhage, Treatment

## Abstract

**Background:**

Gastritis cystica polyposa (GCP) is a recently recognized entity histologically characterized by hyperplasia and cystic dilatation of the gastric glands spreading through the submucosal layer. Its symptoms include those affecting the upper gastrointestinal tract, such as upper abdominal pain, nausea, and anorexia, although some patients might be asymptomatic. GCP rarely causes severe hemorrhage. Recently, we encountered a GCP case that exhibited severe hemorrhage.

**Case presentation:**

A 53 year-old man visited the emergency department complaining of hematemesis. He underwent distal gastrectomy and Billroth II reconstruction for duodenal ulcers 32 years ago. Upper gastrointestinal endoscopy detected bleeding from the reddened mucosa at the anastomosis; thus, tentative endoscopic hemostasis was conducted. Despite medical treatment with transfusion, melena with significant hemodynamic impairment persisted. He was treated again with endoscopic hemostasis and interventional radiology (IVR) but remained unresponsive to these procedures. He eventually underwent partial resection of the anastomosis site with Roux-en-Y reconstruction and finally achieved excellent postoperative recovery. Histopathological examination of the resected specimen suggested a GCP bleeding.

**Conclusions:**

GCP can indeed cause severe hemorrhage. Hemorrhage caused by GCP may not respond to endoscopic hemostasis or IVR; therefore, surgical treatment should be decided without delay.

## Background

Gastritis cystica polyposa (GCP) is a disease proposed by Littler and Gleibermann in 1972, which is a hypertrophic lesion that affects mostly the residual stomach after gastric surgery [1]. Since then, several cases have been reported. Disease terms that similarly describe GCP pathology include gastritis cystica profunda proposed by Franzin et al. [2] and stomal polypoid hypertrophic gastritis proposed by Koga et al. [3]. The reflux of duodenal fluid, which includes bile acids and pancreatic juice, into the remnant stomach as a chemical stimulus is an important factor in GCP formation because GCP is more frequently reported in patients who previously underwent gastric surgery with Billroth II reconstruction.

Macroscopically, GCP often presents as a broad-based polypoid lesion, giant gastric mucosal fold, or submucosal lesion. Recently, GCP has been associated with early or small cancerous lesions. Clinical symptoms of patients with GCP include upper abdominal pain, nausea, and anorexia, as well as abdominal distension caused by gastrointestinal obstruction, although some may be asymptomatic. In rare cases, GCP causes severe hemorrhage. To our knowledge, only few cases of severe anemia or shock associated with massive bleeding in GCP have been reported in the literature. Furthermore, the pathogenesis leading to massive hemorrhage remains unclear. Here, we report a case of GCP that caused severe hemorrhage and required surgical treatment, along with a detailed histopathological examination.

## Case presentation

**Fig. 1:**
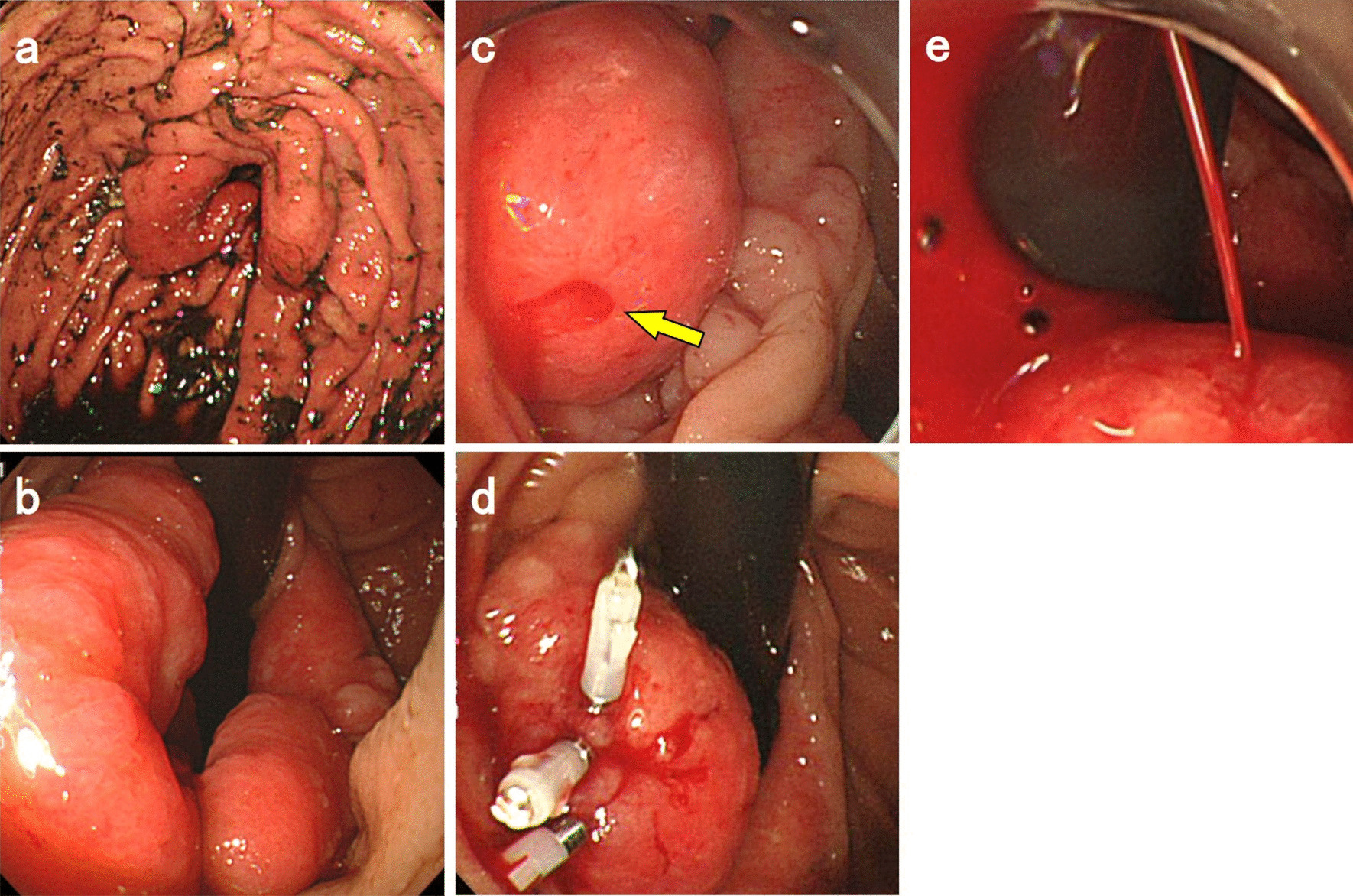
Endoscopic images. **a**, **b** In the first endoscopic view, the source of bleeding was unclear. **c**, **d** Endoscopy performed on the 3rd day of hospitalization. Blood flow (arrow) from the reddened mucosa at the anastomosis site was observed; thus, temporary endoscopic hemostasis by clipping was conducted. **e** When endoscopy was performed again on the 4th day of hospitalization, vigorous bleeding was observed

A 53 year-old man with hematemesis consulted the emergency department. He underwent distal gastrectomy and Billroth II reconstruction for duodenal ulcers 32 years ago. He had bronchial asthma and had been taking steroids for a long time. He had a history of relatively high alcohol consumption, and he smoked 15 cigarettes a day. A general examination revealed that he had anemia. The pulse rate was 126 beats per minute, and the blood pressure was 92/46 mmHg. Regarding his laboratory test, his hemoglobin and blood urea nitrogen levels were 12.1 g/dL and 29.0 mg/dL, respectively, with negative *Helicobacter pylori* infection serologically. Although a large amount of blood clot was found in the remnant stomach, the source of bleeding could not be determined in the first emergency upper gastrointestinal endoscopy (Fig. 1a, b). On the 3rd day of hospitalization, melena was observed; hence, follow-up endoscopy was performed, which revealed bleeding from the reddened mucosa at the anastomosis site, and tentative endoscopic hemostasis was performed by clipping the bleeding site (Fig. 1c, d). Despite medical treatment with transfusion, upper gastrointestinal bleeding symptoms, including hematemesis, melena, and progression of anemia, persisted with significant hemodynamic impairment. On the 4th day of hospitalization, vigorous bleeding was observed when endoscopy was repeated (Fig. 1e). Because it was difficult to secure a suitable endoscopic view due to bleeding, we decided that performing endoscopic hemostasis was impossible and switched to interventional radiology (IVR). The bleeding had subsided spontaneously when IVR was performed, and re-bleeding was not observed during the IVR procedure although endoscopic stimulation was applied to the predicted bleeding site. Arterial embolization with coiling was performed prophylactically on the distribution area, which was predicted as the bleeding site, using an endoclip as a landmark. However, bleeding symptoms persisted, requiring a total of > 20 units of packed red blood cell transfusion. Based on discussions among physicians, surgeons, and radiologist, surgical treatment was necessary because of the uncertainty whether endoscopic hemostasis and IVR could achieve hemostasis and concerns regarding the deteriorating general conditions caused by frequent severe bleeding. With a written informed consent provided, the patient underwent partial resection of the anastomosis site with Roux-en-Y reconstruction. Consequently, he achieved excellent postoperative recovery.

**Fig. 2:**
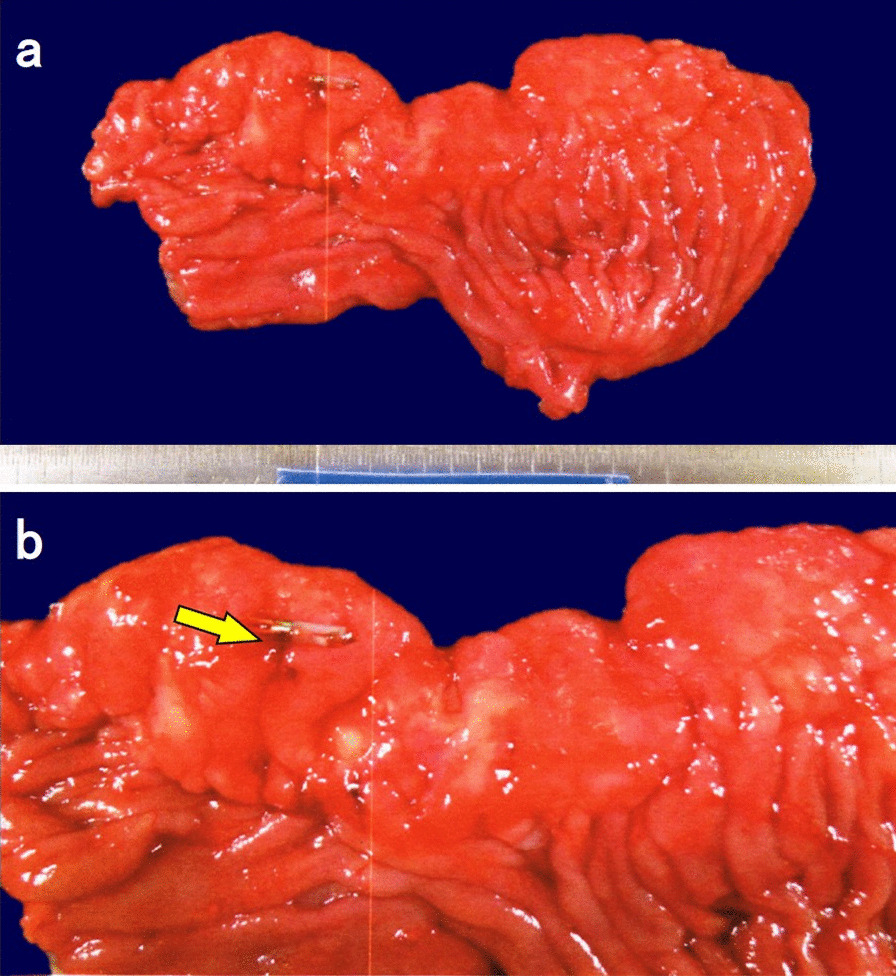
The excised specimen. **a** Macroscopic examination of the excised specimen showed a caterpillar-shaped elevated lesion with a maximum diameter of 70 mm on the greater curvature of the remnant stomach. **b** Some of the elevated lesions had erosion (arrow), which was located near the endoclip and was considered as the bleeding site

**Fig. 3:**
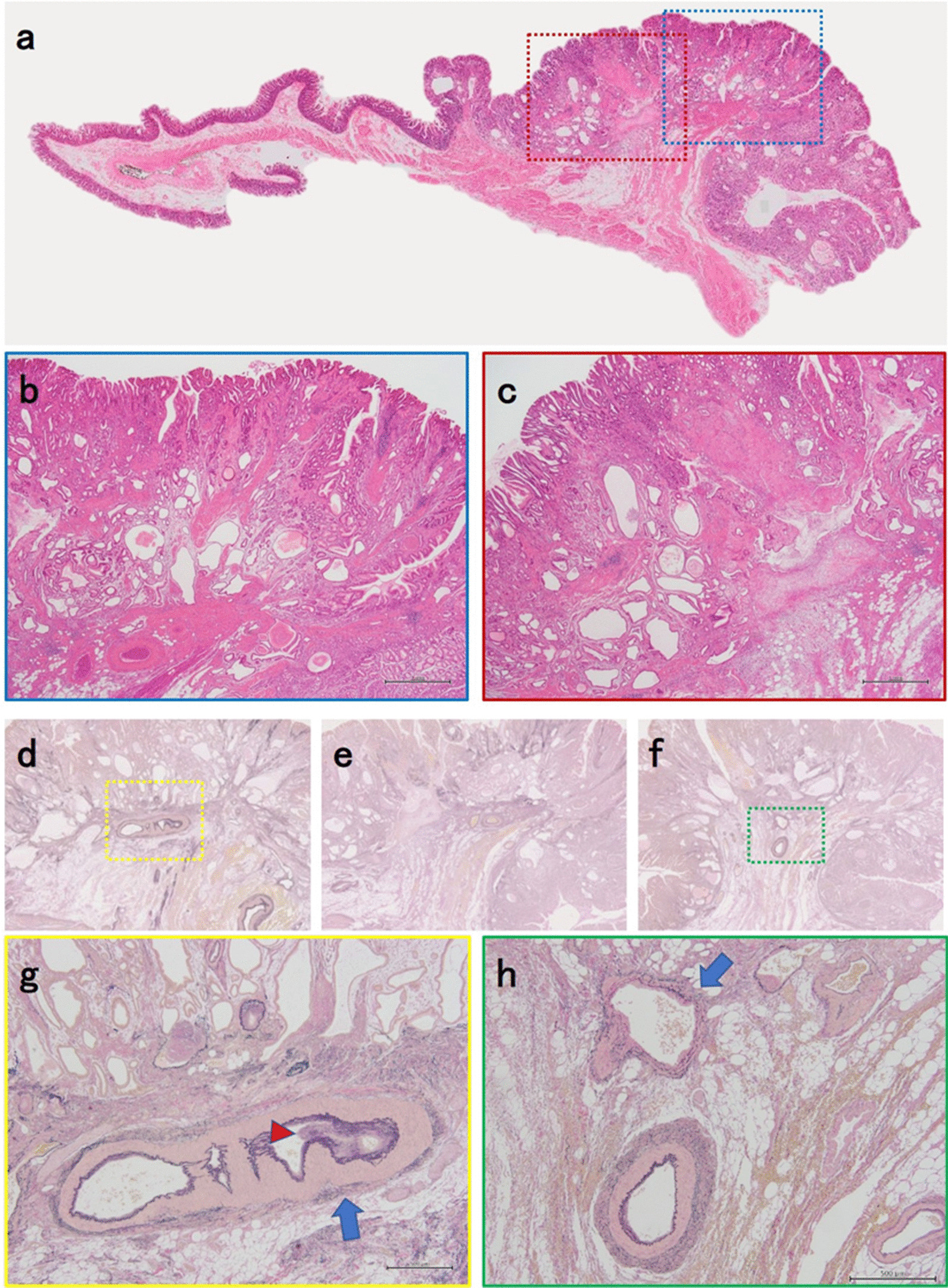
Histopathological view of the resected specimen near the site of endoclip attachment. **a** HE-stained weakly magnified image revealed thickening of the mucosa and hyperplasia of the crypt epithelium. **b** Magnified image of the blue square showed pseudopyloric gland proliferation with cystic dilation in the mucosa. The muscularis mucosa was entangled and elevated radially. **c** Magnified image of the red square showed mucosal damage such as fibrin exudation along with necrosis. No atypical cells were found in the resected specimen. **d**–**f** Histopathological views of the serial resected specimens including the section shown in (**a**). Elastica van Gieson-stained weakly magnified images showed muscular vessels that developed between muscularis and submucosa. **g**, **h** Magnified images of the yellow and green squares showed muscular vessels with an irregularly thickened lumen (arrows). Such lumen was occluded by fibrous tissue (arrowhead)

Histopathological examination revealed the presence of massive hemorrhage. The resected specimen was macroscopically examined, showing a caterpillar-shaped elevated lesion with a maximum diameter of 70 mm on the greater curvature of the remnant stomach. Some of the elevated lesions had erosion, which was observed near the endoclip and was considered as the bleeding site (Fig. 2a, b). Histopathological findings of the resected specimen detected thickening of the mucosa and hyperplasia of the crypt epithelium, as shown in a weakly magnified image (Fig. 3a). In the highly magnified image, the mucosa exhibited pseudopyloric gland proliferation with cystic dilation. The muscularis mucosa was entangled and elevated radially into the ridge, and glandular structures penetrated into the submucosal layer from the gap. Hence, a histopathological diagnosis of GCP was considered. We also observed mucosal damages, such as fibrin exudation along with necrosis. Moreover, the resected specimen had no atypical cells (Fig. 3b, c). However, Elastica van Gieson-stained images showed abnormal muscular vessels that had developed with an irregularly thickened lumen occluded by the fibrous tissue (Fig. 3d–h). Although we could not detect the culprit blood vessels that triggered massive hemorrhage pathologically, we believe that the blood vessel occlusion caused by fibrous tissue buildup resulted from massive bleeding.

## Discussion and conclusions

The etiology of GCP was probably chronic inflammation and ischemia occurring at the anastomotic site postoperatively. However, in some cases, GCP developed in patients with no surgical history [4–7], and the actual etiology remains to be poorly understood. The characteristic histological findings of GCP include crypt epithelium extension while meandering, atrophy of the fundic glands, hyperplasia and cystic dilatation of the pseudopyloric glands, and penetration into the submucosa through the intricate muscularis mucosa. All of these findings were observed in the current case.

GCP is still regarded as a rare diagnosis, and it exhibits nonspecific morphology described as broad-based polypoid lesion, giant gastric mucosal fold, or submucosal lesion. Confirming the diagnosis by the histological examination of biopsy specimens obtained with endoscopic forceps is generally challenging. Therefore, diagnosis confirmation through the histopathological findings of excised specimens, either surgically or endoscopically, is common.

Laratta et al. conducted a review of 37 previously reported cases and reported that 29 men (78%) and eight women (22%), with an overall mean age of 60.5 years (39–81 years), had GCP [8]. Abdominal pain was the most common clinical manifestation (27%), followed by incidental identification (19%) by endoscopy or radiological images, bleeding/anemia (16%), bloating caused by gastrointestinal obstruction (8%), and anorexia/weight loss (8%). Moreover, 65% of the patients had prior gastric resection. To our knowledge, as summarized in Table 1, only six cases of severe hemorrhage associated with GCP have been documented in the literature [1, 9–13]; all cases were male who had undergone prior gastric resection, with a mean age of 50.7 years (13–75 years). Regarding the reconstruction methods of prior gastric surgery, four cases had Billroth II reconstruction, which was described as post gastroenterostomy in two cases. Hemostasis was achieved surgically in three cases, endoscopically in one case, and spontaneously by surveillance in two cases. Of the three surgical cases, endoscopic hemostasis was unsuccessful in one case, while no data was available for endoscopic treatment in two cases. In the present case, the patient was a 53 year-old man who underwent distal gastrectomy with Billroth II reconstruction 32 years ago for duodenal ulcers and recently experienced severe hemorrhage caused by GCP. Partial resection of the anastomosis site with Roux-en-Y reconstruction was conducted because endoscopic hemostasis and IVR treatment were ineffective.

**Table 1:** Profiles of reported cases of severe hemorrhage with gastritis cystica polyposa in the literature

No	References	Age	Sex	Reconstruction method of prior gastric surgery	Treatment for hemostasis
1	Littler et al. [1]	47	M	Gastroenterostomy	Partial gastrectomy with gastroenteric anastomosis
2	Griffel et al. [9]	69	M	Billroth II	Partial gastrectomy with Roux-en-Y reconstruction
3	Kurland et al. [10]	75	M	Billroth II	Endoscopic hemostasis
4	Itte et al. [11]	50	M	Gastroenterostomy	Exploratory laparotomy
5	Ali et al. [12]	13	M	Billroth II	Surveillance
6	Tominaga et al. [13]	50	M	Billroth II	Surveillance
7	Present case	53	M	Billroth II	Partial gastrectomy with Roux-en-Y reconstruction

The mechanism of massive hemorrhage caused by GCP is still unclear. Koga et al. reported that 25 (66%) of the 38 patients who underwent resection after gastrectomy had localized thickening of the gastric mucosa at the anastomosis, which exhibited a series of changes from mild to severe both macroscopically and histologically [3]; therefore, chronic anastomosis stomatitis might be a factor of polyp-like elevations. Localized stomach mucosal hyperplasia and submucosal ectopic glands and cysts may happen at the sites of repeated erosion and regeneration caused by various stimuli. Moreover, Juler et al. described that vascular dysplasia can be caused by chronic inflammation, and conditions associated with abnormal blood vessels, such as vascular dysplasia, arteriovenous malformation, and dilated blood vessels that appear during the process of GCP development and proliferation, are highly probable [14].

In the present case, the histopathological findings of the resected specimen revealed abnormal muscular vessels along with several mucosal damages, consistent with the GCP thick-walled vessels reported by Giffel et al. [9]. Consequently, the specific exposed blood vessels that caused bleeding could not be identified, but we hypothesized that mucosal damage might be repeated because of various chronic stimuli, such as duodenal fluid reflux, which eventually leads to massive hemorrhage because of damaged abnormal vessels. In addition, the patient was taking steroids for a long time, which might have led to tissue fragility and intractable bleeding.

GCP is generally considered as a benign lesion; however, it has been recently associated with early or small cancerous lesions [4–6]. Its malignant potential remains controversial, and the incidence of GCP-associated gastric cancer is still unclear. Moreover, it might be difficult to distinguish GCP from gastric cancer. In the present case, no atypical tissue was detected.

Considering that this report is a case study, it has limitations. GCP is still a rare disease, and comprehensive reports about GCP have remained unavailable. Thus, its pathophysiology, natural history, and malignant potential are still poorly known. We hope that these aspects will be elucidated by future studies, and more evidence-based follow-up methods and treatment strategies will be established.

To conclude, GCP can cause severe hemorrhage, which may not respond to endoscopic hemostasis or IVR, surgical treatment needs to be decided without delay. We recommend careful endoscopic follow-up of anastomotic lesions such as GCP because precancerous features cannot be ruled out and can lead to anemia and massive bleeding.

## Data Availability

This case report contains clinical data from the electronic medical record of the Kokuho Central Hospital. The information is available from the corresponding author or the editor on reasonable request.

## Abbreviations

GCP: Gastritis cystica polyposa
IVR: Interventional radiology
